# Priorities for the development of alfalfa pasture in northern China

**DOI:** 10.1016/j.fmre.2022.04.017

**Published:** 2022-05-02

**Authors:** Tianzuo Wang, Wen-Hao Zhang

**Affiliations:** State Key Laboratory of Vegetation and Environmental Change, Institute of Botany, the Chinese Academy of Sciences, Beijing 100093, China

**Keywords:** Alfalfa (*Medicago sativa* L.), Breeding, Field management, Harvest, Forage processing

## Abstract

Alfalfa (*Medicago sativa* L.) is acclaimed as “Queen of forages” because of its great yield and high feeding value. China is the second biggest country in acreage of alfalfa cultivation, but the cultivation regions of alfalfa are distinguished by adverse climatic and edaphic conditions in northern China. Moreover, the lack of elite alfalfa varieties with great adaptation and poor field management are vital factors limiting development of alfalfa pasture in China. In addition, nutritional quality of alfalfa in China is also poor compared to that in countries of developed animal husbandry industrial. Here, we propose several priorities in terms of a comprehensive system of alfalfa breeding, field management, harvest and processing with Chinese characteristics, based on the theories and methodologies of breeding science, agronomy, plant physiology and agricultural machinery. Implementation of these priorities will greatly contribute to the sustainable development of the alfalfa pasture in China.

## 1 Introduction

Artificial pasture, particularly alfalfa pasture, has tremendous advantages in yield and nutritional quality of forage over natural grassland. The acreage, production level and management of artificial pasture are key criteria to determine the developmental degree of animal husbandry in a country and/or region. The total area of grassland in China is about 280–393 million hectares, accounting for 30–40% of the land area. However, the proportion of artificial pasture to the total grassland in China is only ca. 5%, far less than that in New Zealand (70%), UK (59%) and USA (15%) [1]. Therefore, the development of artificial pastures with great yield and high nutritional quality is an effective means to meet the demands of forage for animal husbandry in China.

**Fig. 1:**
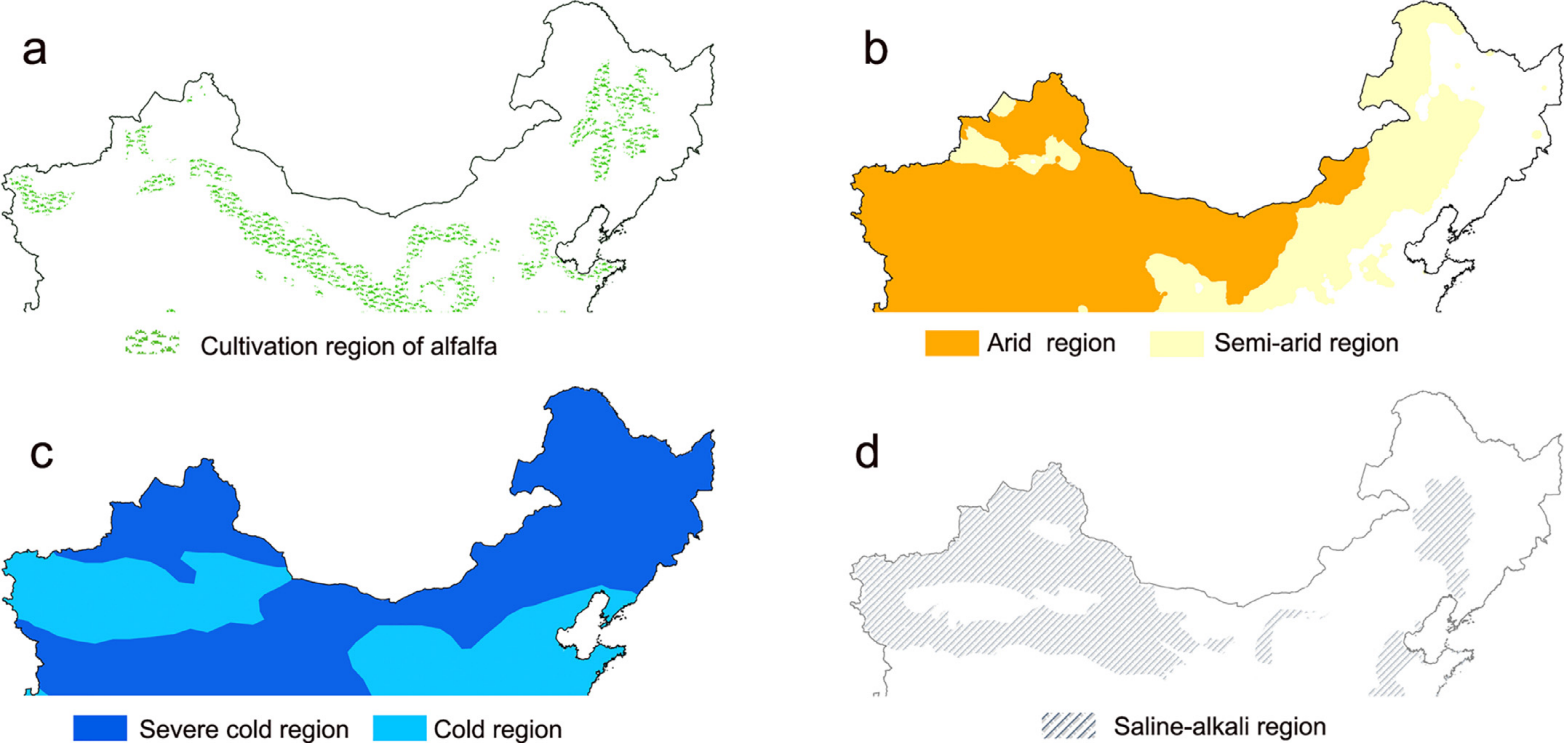
**The climatic and edaphic conditions of the alfalfa cultivation regions in northern China.** (a) Overall cultivation regions of alfalfa. (b) Arid and semi-arid regions. (c) Severe cold and cold regions. (d) Saline-alkali regions. These figures were drawn based on the standard map of China (No. GS (2016) 1570).

Alfalfa (*Medicago sativa* L.) is the most important perennial legume forage worldwide and has been cultivated in more than 80 countries [2]. Alfalfa is often referred to as the “Queen of forages” because of its great forage yield, high contents of protein, vitamins and minerals. Despite long history of alfalfa cultivation in northern China, the large scale cultivation of alfalfa has been increased markedly since 2010 due to the Melamin event in 2008 [3]. China has now become the second biggest country in terms of alfalfa acreage. However, the unfavorable climatic and edaphic conditions, such as drought, low temperature and soil saline-alkali, in the main cultivation regions of alfalfa in China have great negative impacts on yield and quality of alfalfa in China (Fig. 1). Moreover, the lack of elite alfalfa varieties and poor field management are also critical factors limiting development of alfalfa pasture in China. The yield of alfalfa forage per hectare in China is less than in those countries (e.g., USA and New Zealand) with advanced animal husbandry due mainly to the negative effects. In addition to the low forage yield, the nutritional quality of alfalfa in China is also low compared to that in the countries of developed animal husbandry industrial. The low alfalfa yield and nutritional quality cannot meet the increasing demand of the rapid development of animal husbandry in China. To solve this problem, a great amount of high-quality alfalfa hay has been imported to China. For example, alfalfa hay imported into China has increased from ca. 200,000 to 1400,000 tons from 2010 to 2020, making China the biggest importer of alfalfa hay [4]. This trend will be continued in the near future. To successfully establish the alfalfa pasture in northern China, particularly in the farming-pasture ecotone, we propose several priorities, which can also provide valuable references for those countries in a similar situation with China, particularly the Belt and Road countries.

## 2 Alfalfa breeding

The number of alfalfa varieties approved officially in China is much less than that in the countries with developed animal husbandry industry. For example, only about 110 alfalfa varieties have been approved and registered in China since 1986, with an average of about 3 varieties being registered annually. In contrast, more than 60 alfalfa varieties are registered in the USA annually. The alfalfa varieties have to be long-term used in practice in China because of the lack of alfalfa varieties, leading to the degeneration of varieties in terms of yield and nutritional quality. Accordingly, many foreign alfalfa varieties have been introduced to China. Alfalfa is mainly grown in northern China with arid climate (Fig. 1b), cold winter (Fig. 1c), and infertile soil. In some areas of Xinjiang, Gansu, Songnen Plain of Northeast and Yellow River Delta, soil saline-alkali is also an important factor restricting alfalfa yield and quality (Fig. 1d) [5]. The introduced foreign alfalfa varieties often exhibit high forage yield, but have less plasticity to adverse environment.

In the long-term domestication progress of cultivated alfalfa, great attention has been paid to the traits associated with high forage yield, leading to loss of numerous genes involved in tolerance of alfalfa to environmental stress. On the other hand, there are many wild legume forages native to natural ecosystems in northern China with distinct adaptive characteristics to the harsh environments. Of them, *M. falcata* and *M. ruthenica* are two close relatives of alfalfa, which exhibit outstanding tolerance to environmental stress compared to alfalfa, thus providing valuable genetic resources for breeding alfalfa varieties with high tolerance to environmental stress [6]. Indeed, these resources have been used as parental materials to breed new alfalfa cultivars with strong tolerance and high yield. For example, the alfalfa varieties Longmu 803 and Caoyuan No. 3 that have been widely planted in northern China are bred by hybridization with *M. ruthenica* and *M. falcata*, respectively. In addition, an increasing number of quantitative trait loci (QTL) and genes responsible for tolerance to harsh environments are identified and characterized by wild legume forage with the development of advanced technology in molecular biology. These studies not only identify the specific tolerant pathways absent in the alfalfa varieties, but also provide the theoretical basis to improve the traits associated with tolerance to environmental stress by genetic breeding [7].

**Fig. 2:**
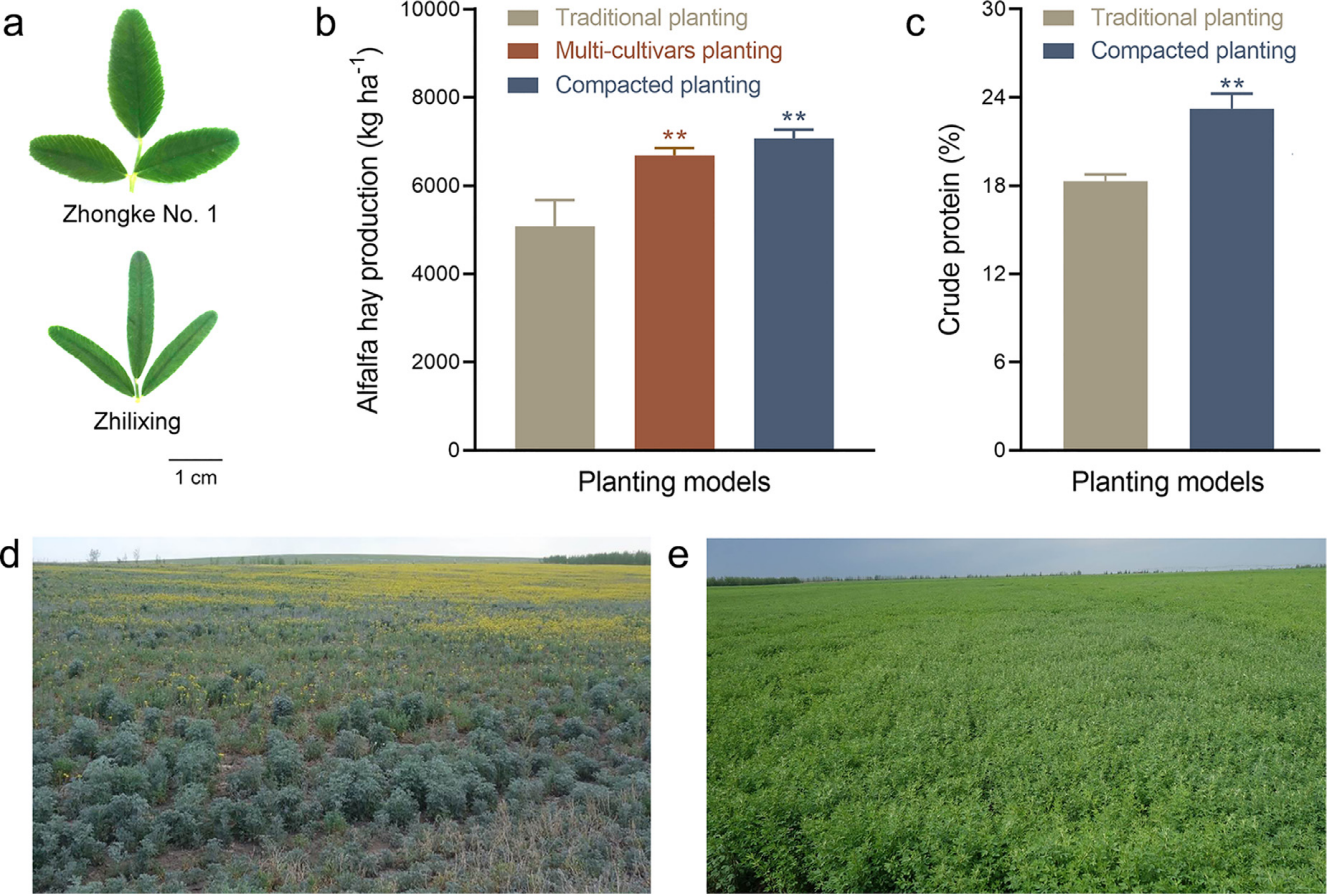
**The case examples of *Medicago* breeding and alfalfa pasture management.** (a) Leaf size comparison of *M. ruthenica* ‘Zhongke No. 1’ and ‘Zhilixing’. (b) Alfalfa hay production of different planting models. (c) Crude protein concentration of different planting models. (d) Traditional planting model of alfalfa pasture. (e) Compact planting model of alfalfa pasture. Hay production and protein concentration of alfalfa hay were based on the data of 2017, 2018 and 2019. Photos of traditional and compact planting models were taken in 2017. ** means significant difference between the different planting models at *P*<0.01.

Alfalfa hay quality is mainly determined by its crude protein concentration. The protein concentrations in alfalfa leaves are significantly higher than those in stems. Therefore, breeding alfalfa varieties with high leaf/stem ratios can be an effective way to improve nutritional quality. In this context, *PALM1* that encodes a zinc finger transcription factor has been identified to play an important role in the control of leaf morphogenesis, such that mutation of *MsPALM1* led to five leaflets per leaf in alfalfa, compared with three leaflets per leaf of the wild-type [8], thus improving the quality of the mutants. In addition to an increase in the number of leaflets, breeding new alfalfa varieties with large leaves may be another way to improve the quality of alfalfa due to higher protein concentration in leaves. In this context, a new variety of *M. ruthenica* (Zhongke No. 1) with enhanced protein concentration resulting from large leaves was bred (Fig. 2a). In addition to protein concentration, concentrations of cellulose and lignin are other important factors in determining forage quality. High concentrations of cellulose and lignin are not conducive to the digestion and absorption of forage by livestock. A new variety of alfalfa Hi-GEST with low lignin has been developed and used in USA. However, generally speaking, nutritional quality has been paid much less attention in the alfalfa breeding compared to forage yield.

## 3 Management of alfalfa pasture

In addition to selection and breading elite alfalfa varieties with high forage yield and great adaptation to environments, effective management of alfalfa pasture is also an important measure to improve the yield and quality of alfalfa forage. The conditions of alfalfa establishment and management in China differ from those in other countries with developed animal husbandry. Unfortunately, alfalfa pasture managements in China often copy the methods and protocols used in those countries like USA and UK [3]. Therefore, new concepts, methodologies and paradigms that fit the climatic, edaphic and managerial conditions in China are urgently needed.

### 3.1 Multi-cultivars planting model of alfalfa pasture

To maximize and stabilize forage yield as well as optimize resource utilization, we develop a new model of alfalfa planting by mixing several alfalfa varieties with contrasting phenotypes and genetic backgrounds. We found that the maximal and consistent alfalfa hay production was achieved by planting alfalfa with 4 to 6 cultivars in Inner Mongolia, China. Different alfalfa cultivars differ in their shoot and root architectures, allowing them to efficiently utilize light, water and nutrients upon mixing them in alfalfa pasture, such that the hay production is increased compared with the traditional planting model (Fig. 2b). Moreover, the multi-cultivars planting model can enhance the tolerance of the alfalfa population to abiotic and biotic stress due to the diverse genetic background and complexity of the ecosystem.

### 3.2 Compact planting model of alfalfa pasture

In the traditional alfalfa planting patterns, sowing row spacing is about 30–50 cm with a seed sowing rate of 7.5–15 kg ha^−1^. However, we discovered that narrowing the row spacing to 10–15 cm and correspondingly increasing the sowing rate to 15–22.5 kg ha^−1^ can increase the alfalfa hay production and protein concentration by 39% and 27% in the Hulunbuir, Inner Mongolia (Fig. 2b and c). The overall benefit resulting from increased forage production is much greater than the cost associated with increased seed sowing by the compact planting model. Moreover, the compact planting model also effectively controlled weeds and minimized evaporation due to rapid coverage of the land during the early stage of seedling establishment (Fig. 2d and e), thus improving the water use efficiency of the alfalfa pasture.

### 3.3 Fertilizer management of alfalfa pasture

Few studies have specifically focused on nutrient uptake and utilization, as well as fertilizer management of alfalfa pasture compared to those annual grain crops. Substantial differences exist between alfalfa and traditional crops in terms of harvesting objects (aboveground vegetative organs vs grains) and plant growth types (perennial vs annual). Currently, nutrient management of alfalfa pasture is generally copied from that used in annual crops. In addition, nutrient management in alfalfa pasture in China often follows that used in the USA. The uniqueness of alfalfa pasture in China relative to other counties in terms of climatic, edaphic and managing conditions requires the development of nutrient management regimes and policies with distinct Chinese characteristics to guide and support the sustainable development of alfalfa pasture. To address these issues, we should strengthen studies on the nutrient acquisition of alfalfa pasture by specifically focusing on rhizosphere processes. The lack of knowledge on nutrient acquisition by alfalfa pasture also hampers the development of fertilizers specific to alfalfa pasture in China.

### 3.4 The pasture for grazing

In the large areas with relatively harsh environment and fragile ecosystem, development of pasture for grazing has advantages over the traditional pasture for hay production. Development of pasture for grazing can minimize the cost associated with hay harvesting, packing and transportation. Moreover, livestock manure can be directly used as fertilizer for the pasture, which can also reduce the cost for fertilizer application. In addition to promotion of forage production, the pasture for grazing also has the ecological function of protecting water and soil resources. Selection and breeding alfalfa of creeping type with great tolerance to trampling and strong tillering ability is a priority for the development of grazing-type alfalfa pasture.

### 3.5 Development of marginal land

Marginal land refers to the lands with low agricultural productivity, poor economic benefits and fragile ecology due to severely barren soil, strong constraints on water/heat resources and adverse topographic conditions. The area of marginal land in China is approx. 78 million hectares, which is about 3 times larger than that of artificial pasture in China. Compared with traditional crops, alfalfa is more tolerant to the adverse climatic and edaphic conditions in marginal land, thus making it a priority to plant alfalfa in marginal land. Moreover, as a legume, alfalfa can also improve soil fertility and improve degraded grasslands [9]. Therefore, more marginal land can be used for establishing alfalfa pasture, which is an effective way to expand the acreage of alfalfa and improve the ecological environment in the ecologically vulnerable regions.

## 4 Harvest and processing

The low efficiency of highly specified machinery for harvesting and processing alfalfa hay is another critical factor limiting alfalfa production and industry in China. Most alfalfa has to be dried naturally in the field after harvesting due to the shortage of drying machinery in China. It takes a longer time to dry up the harvested alfalfa because of the relatively high humidity and low temperature during natural drying, thus deteriorating alfalfa's quality resulting from loss of leaves and nutrition consumption during the natural drying process. According to statistics, the loss of forage during harvest and drying is as high as more than 20% of total forage production in China, compared with 5% in the countries with developed animal husbandry. Therefore, the development of drying machinery to efficiently process alfalfa hay is urgently needed for the alfalfa industry in China. In addition to the harvest of hay, other types of alfalfa processing, such as silage, grass granule and leaf protein, have much higher accessional values than hay [10], which should also be paid more attention.

**Fig. 3:**
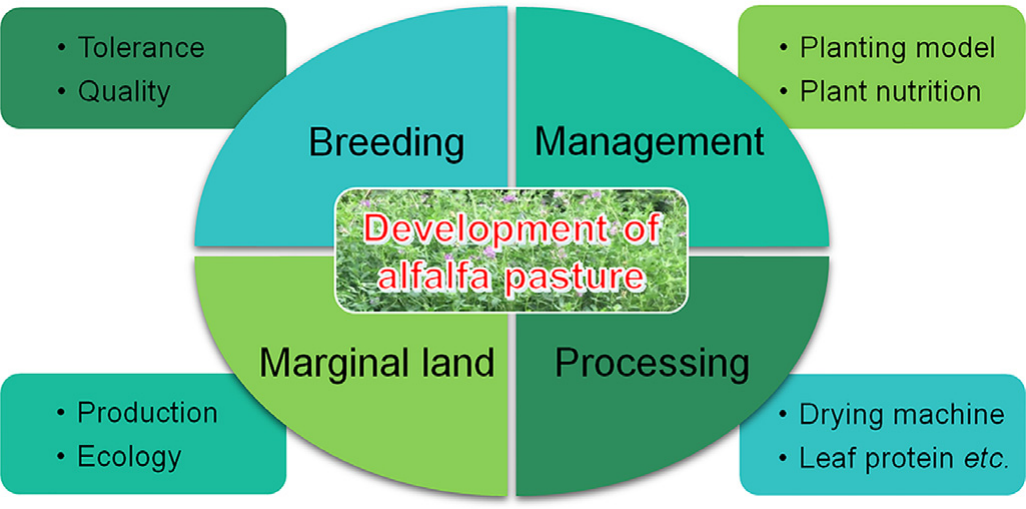
**The proposed priorities for the development of alfalfa pasture in northern China.**

Based on the theories and methodologies of breeding science, agronomy, plant physiology and agricultural machinery, a comprehensive system of breeding, field management, harvest and processing for alfalfa with Chinese characteristics (Fig. 3) will greatly contribute to the sustainable development of the alfalfa pasture in China.

## Declaration of competing interest

The authors declare that they have no conflicts of interest in this work.
